# Patient Satisfaction and Factors Associated With Anesthesia Services in Ethiopia: A Systematic Review and Meta‐Analysis

**DOI:** 10.1002/hsr2.72555

**Published:** 2026-05-25

**Authors:** Basazinew Chekol Demilew, Agazhe Aemro, Habtie Bantider, Diriba Teshome

**Affiliations:** ^1^ Department of Anesthesia, College of Medicine and Health Science Debre Markos University Debre Markos Ethiopia; ^2^ Department of Medical Nursing, College of Medicine and Health Science Debre Markos University Debre Markos Ethiopia; ^3^ Department of Anesthesia, College of Health Science Debre Tabor University Debre Tabor Ethiopia

**Keywords:** anesthesia service, determinants of satisfaction, Ethiopia, patient care, patient satisfaction

## Abstract

**Background and Aims:**

Patient satisfaction is the patient's reaction in the form of a “cognitive evaluation” and “emotional response” to the care they receive. It is one of the indicators of patient care outcomes. Therefore, this study aimed to determine the pooled level of patient satisfaction and factors associated with anesthesia service in Ethiopia.

**Methods:**

This systematic review and meta‐analysis was done by searching studies from databases PubMed/MEDLINE, Google Scholar, and Google by using the PEO criteria. The quality of the studies was appraised by the modified Newcastle–Ottawa assessment tool adopted for cross‐sectional studies. Data were extracted by reviewers independently using Microsoft Excel and then exported to STATA 16 version statistical software for analysis. Heterogeneity and publication bias were assessed using the *I* ^2^ statistic and a funnel plot and Egger test, respectively. The pooled percentage of satisfaction and its associated factors (POR) with a 95% confidence interval was estimated using random model meta‐analysis.

**Result:**

Thirteen cross‐sectional studies with a total of 3277 participants were included in this study. The pooled percentage of patient satisfaction was 75% (95% CI: 65%–84%). Having no postoperative pain, no postoperative nausea/vomiting, being done with a regional anesthesia, and having previous anesthesia exposure were significantly associated with the outcome variable, as explained with AOR and 95% CI of 2.34 (1.95–2.73), 2.41 (1.74–3.07), 2.74 (2.27–3.2), and 2.96 (1.4–4.53), respectively.

**Conclusion:**

According to this study, 75% of patients were satisfied with the anesthesia services they received. Although this suggests a favorable judgment, there is still much room for improvement in perioperative care. The results showed that clinical comfort and the type of anesthetic method employed are the main factors influencing patient satisfaction. Particularly, the absence of postoperative pain and nausea/vomiting, the use of regional anesthesia, and prior anesthesia experience were the determining factors.

**Abbreviations**
CI: confidence interval
OR: odds ratio
PONV: postoperative nausea and vomiting

## 1 Introduction

Perioperative patient care is a practical, demanding, complex, evidence‐based, and innovative approach that requires the essential skills and knowledge of perioperative professionals to care for their patients in a multidisciplinary approach [1, 2, 3]. This patient care includes preoperative, intraoperative, and postoperative processed periods [4]. Preoperative care includes patient evaluation, stratification, preparation, and optimization. Intraoperative patient care incorporates the management of planned anesthesia and all necessary interventions during the time of surgery [5]. Whereas postoperative patient care is all about interventions held starting immediately after the end of surgery.

According to Pascoe's definition, patient satisfaction is the patient's reaction in the form of a “cognitive evaluation” and “emotional response” to the care they receive. It explains how the patients are happy with the services delivered for their care. It is a subjective assessment of the service obtained, considering the individual's expectations, and is a crucial component in determining the quality of healthcare. It may affect a variety of areas of their behavior, including overall resource consumption, adherence to treatments, and the consistency of connection with their caregivers [6, 7, 8].

It is one of the indicators of patient care outcomes, which is affected by many factors such as the socioeconomic status, demographic data, cultural level, patients' preferences, cognitive ability of the patients, past experiences, and quality of the tool used. Assessing the level of patient satisfaction is vital to improving the quality of care in perioperative anesthesia services. Anesthetist‐to‐patient interaction, perioperative anesthetic management, and postoperative follow‐up may affect patient satisfaction [2, 9, 10].

Knowing the level of patient satisfaction with anesthesia service may help to improve the way patient care is delivered. Anesthesia services are essential to patient safety and comfort in the field of perioperative medicine, but perceptions of this care in settings with limited resources, such as Ethiopia, are inconsistent across different studies. Although some cross‐sectional reports have tried to measure satisfaction levels, their results differ greatly because of variations in patient demographics and institutional protocols. Also, evidence regarding the determinants of patient satisfaction was variable across the country. For the improvement of anesthesia care, it is better to go from fragmented evidence to more national and aggregated evidence for policy making and planning of activities for the quality of anesthesia care. Therefore, this systematic review and meta‐analysis aimed to determine the pooled percentage of patient satisfaction and factors associated with anesthesia service in Ethiopia.

## 2 Methods

### 2.1 Study Setting and Search Strategies

This systematic review and meta‐analysis was conducted to estimate the pooled satisfaction level and associated factors of patients who underwent surgery under anesthesia in Ethiopia. Potential studies done in Ethiopia were identified using databases PubMed/MEDLINE, Hinari, Google Scholar, and Google Search. Additionally, a hand search was applied to identify additional literature by using key terms and via cross‐references, links, and citations. All searches were limited to the English language and studies published within 10 years from Ethiopia. The search was performed on April 25–30, 2025 from all databases. We searched PubMed with Medical Subject Headings (MeSH) terms and direct searching with terms, not MeSH. The terms used during direct searching of PubMed were “(patient OR mothers) AND (anesthesia service OR perioperative care) AND (satisfaction).” Whereas the MeSH terms used were (((((((((patient) OR (maternal)) OR (mothers)) OR (parturient)) OR (elective cases)) AND (anesthesia service)) OR (preoperative evaluation)) OR (postoperative pain management)) OR (anesthesia)) AND (satisfaction). The results were further restricted by free full text and the human species. Also, gray literature was searched with the search term “Patient Satisfaction and factors associated with Anesthesia service; Ethiopia” to not miss important articles. This systematic review and meta‐analysis was prepared and reported with the PRISMA checklist. The registration was submitted for Prospero registration [11].

### 2.2 Eligibility Criteria

We used *PEO* (*Population*: Patients who underwent surgery under anesthesia; *Exposure*: Anesthesia service; *Outcome*: Patient satisfaction) approach to include and exclude studies.

#### 2.2.1 Inclusion Criteria

This systematic review and meta‐analysis included articles that met the following criteria:

*Study design:* All types of studies (cross‐sectional, case‐controls, and cohort, etc.).

*Language:* The articles were published only in the English language.

*Population:* Patients who had exposed for any type of anesthesia service.

*Publication condition:* Both published and unpublished articles from different universities' repositories.

*Publication year:* All publications reported since 2014 G.C.

*Exposure:* Anesthesia services; preoperative assessment and preparation, intraoperative care, and postoperative care and monitoring.

*Outcome:* Satisfaction and factors associated with anesthesia service.

*Setting:* studies done in Ethiopia.

#### 2.2.2 Exclusion Criteria

Studies that lacked acceptable data or had incomplete data were eliminated from this analysis. Furthermore, studies with no complete text and no response from the respective authors to obtain the full text were removed from this meta‐analysis.

### 2.3 Outcome Measurement

The main outcomes of interest for this meta‐analysis were the pooled level of satisfaction and associated factors among patients who underwent surgery under anesthesia.

### 2.4 Quality Assessment and Data Extraction

The quality of the studies was critically appraised by the modified Newcastle–Ottawa appraisal assessment tool established for cross‐sectional studies [12, 13]. The qualities of each study were weighted by all authors (B. C. D., A. A., H. B., and D. T.) individually using the quality assessment tool criteria. Those primary studies with a medium score (satisfying 50% quality evaluation criteria) and high quality (≥ 7 out of 10) were included in this study. The investigators' differences were managed by taking the average score of their quality evaluation outcomes. The quality of all the included 13 studies was graded as stated in the table (Table 1).

**Table 1:** Characteristics of cross‐sectional studies included in the systematic review and meta‐analysis of the level of satisfaction for patients who underwent surgery under anesthesia.

First author, publication year	Study area	Study population	Sample size	Satisfaction (%)	Quality of study
Samuel D. et al., 2020 [14]	Ethiopia, Gondar	Parturients	383	82.3	High
Yosef B. et al., 2020 [15]	Ethiopia, Gondar	All groups of patients	418	72.2	High
Kore M. et al., 2019 [16]	Ethiopia, Mekelle	All groups of patients	120	88.33	High
Basazinew D. et al., 2021 [17]	Ethiopia, Debre Tabor	Parturients	120	80.2	High
Atsedu S. et al., 2021 [6]	Ethiopia, Gondar	All groups of patients	398	74	High
Melaku B. et al., 2022 [18]	Ethiopia, Multicenter	All groups of patients	411	64	High
Endale G. et al., 2015 [19]	Ethiopia, Gondar	All groups of patients	156	90.4	High
Endale G. et al., 2014 [5]	Ethiopia, Gondar	All groups of patients	102	65	Moderate
Mohammed S. et al., 2017 [20]	Ethiopia, Menelik II Hospital	All groups of patients	224	72.3	High
Amanu G. et al., 2020 [21]	Ethiopia, Hawassa	All groups of patients	200	60	High
Diriba T. et al., 2022 [22]	Ethiopia, Debre Tabor	All groups of patients	387	62.3	High
Abayneh et al., 2015 [23]	Ethiopia	All groups of patients	183	99.3	High
Hagos et al., 2017 [24]	Ethiopia	Parturients	175	62	High

A Microsoft Excel spreadsheet with the authors' names, year of publication, study area, study design, sample size, level of satisfaction, and factors with AOR and 95% CI was used as a data extraction tool. Furthermore, an information extraction framework was developed for each unique linked factor that was significantly associated with the primary outcome of the included studies. The titles and abstracts of all identified literature in the searches were reviewed by two authors (B. C. D. and D. T.). Included studies were reviewed by two authors independently, and decisions were made regarding selection/rejection. The disagreements arising were resolved by the discussion of all the authors.

### 2.5 Statistical Analysis

The extracted data were imported to STATA^MP^ version 17.0 software for analysis. After checking the heterogeneity of included studies, the pooled prevalence of patient satisfaction and associated factors was determined by the random‐effects model using the DerSimonian–Laird method [25]. The results were presented using texts, tables, and different plots with measures of effect and a 95% confidence interval. Meta‐regression, subgroup analysis, Egger's test, and the trim and fill test were done.

#### 2.5.1 Heterogeneity and Publication Bias

The *I* ^2^ statistic was used to evaluate the presence or absence of heterogeneity between studies [25]. Subgroup analysis by using sample size and publication year was performed to minimize heterogeneity. Sensitivity analysis was done to determine the possible included outlier articles. Publication bias was checked by using a funnel plot and Egger test [26, 27].

## 3 Results

### 3.1 Study Selection Process

A thorough search of various sources was the first step in identifying possible researches.

Initially, databases and registers yielded a total of 8970 records. Although PubMed (4940 records) and Google Scholar (524 records) provided the majority of records, Science Direct, AJOL, and DOAJ provided additional records. Also, six records were found using “other methods,” like citation searching (three records) and organizational repositories (three records). Theses pool was then thoroughly filtered as: 4872 duplicates being eliminated, 3497 being marked as ineligible, and 466 being eliminated for various reasons.

Following the initial cleanup, a more detailed manual screen was done. A total of 153 records from the database pool were screened and 15 of these were excluded. Of the remaining 138 reports sought for retrieval, a majority (123 records) could not be retrieved, leaving only 15 articles for the eligibility assessment. Out of the six records found via other methods, only one report was successfully sought and retrieved for eligibility assessment.

**Figure 1:**
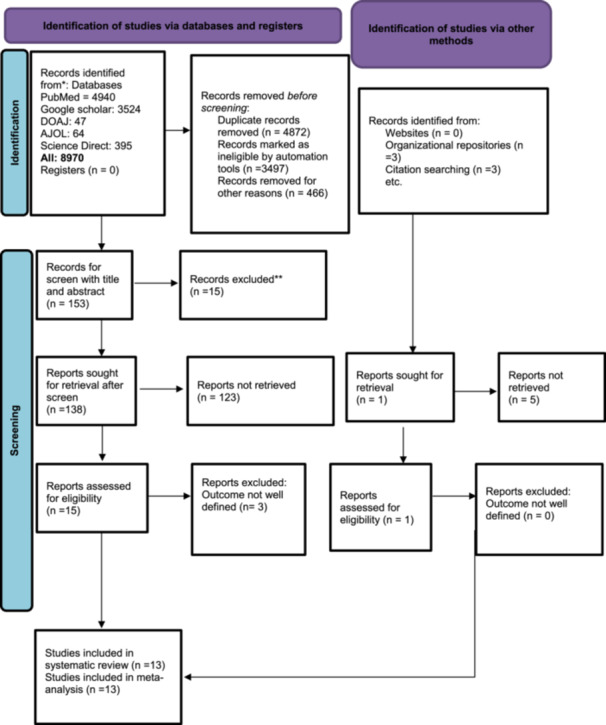
PRISMA flow diagram showing search strategies and results.

Finally, the full text of the retrieved reports was assessed against specific criteria. From the 15 reports assessed from databases, 3 were excluded because the “Outcome was not well defined.” From the 1 report assessed from other methods, it passed the eligibility check with 0 exclusions. The final result of this rigorous filtering process led to 13 studies being included in the final systematic review and meta‐ analysis process (Figure 1) [11].

### 3.2 Characteristics of Included Studies

In the current systematic review and meta‐analysis, a total of 13 studies were included with a sample size ranging from 102 [5] to 418 [15]. The proportion of patient satisfaction among the included studies varied from 60% [21] to 99.3% [23]. Regarding study design and setup, all included studies employed a cross‐sectional study design done in Ethiopia. Furthermore, concerning the study population, 3 studies were conducted only on parturients [14, 17, 24] whereas the remaining 10 studies were done on all groups of patients [5, 6, 15, 16, 18, 19, 20, 21, 22, 23]. From those included studies, seven studies are used for the extraction of data for factor analysis [6, 14, 15, 17, 18, 21, 22]. The remaining six papers do not have enough information for the association of factors [5, 16, 19, 20] (Table 1).

### 3.3 Meta‐Analysis

#### 3.3.1 Publication Bias

**Figure 2:**
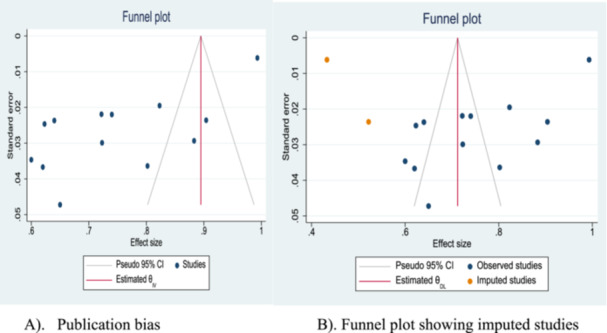
Funnel plot to test publication bias of included studies and show the missed studies. (A) Publication bias. (B) Funnel plot showing imputed studies.

The possibility of publication bias across the studies was observed by using a funnel plot and Egger's regression test [27, 28]. The funnel plot and Egger's test indicated that there is publication bias observed between the studies (Egger's regression tests *p* values of 0.0139). The asymmetry of the funnel plot also indicated that there is publication bias (Figure 2A). The trim and fill statistics showed there are two imputed studies identified for publication bias, and the funnel plot also indicated the missed studies (Table 2 and Figure 2B).

**Table 2:** The trim and fill results of the included and missed articles.

Studies	Number of studies	Effect size	95% CI
Observed	13	0.749	0.654–0.844
Observed + imputed	2 + 13	0.712	0.564–0.86

### 3.4 The Pooled Level of Patient Satisfaction for Anesthesia Service

**Figure 3:**
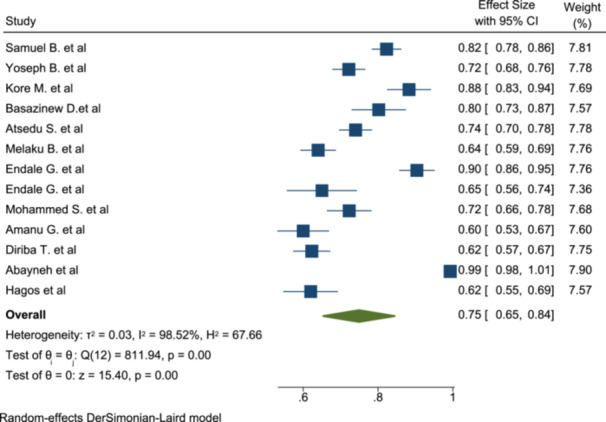
Forest plot showing the pooled estimate of patient satisfaction with anesthesia service.

A total of 13 studies with 3277 participants were included to determine the pooled level of satisfaction for anesthesia service of patients who underwent surgery under anesthesia. In the included studies, satisfaction rates varies from 60% [21] to 99.3% [23]. According to this meta‐analysis, the pooled patient satisfaction rate for anesthesia services is 75%, with a 95% confidence interval of 65%–84%. We employed the random effect model to determine the pooled level of satisfaction because of the significant degree of heterogeneity across the included studies (*I* ^2^ = 98.5%, *p* = 0.00) (Figure 3).

### 3.5 Subgroup Analysis

Subgroup analysis was performed by study population sample size (> 300 vs. 300) and publication year (published by 2020 and later vs. before 2020) to find possible causes of heterogeneity for included studies. Accordingly, the lowest and highest satisfaction rate was found from the publication year since 2020 to be 70.8% (95% CI 64.4–77.2) and 79.9% (95% CI 67.2–92.5) for the year before 2020 (Table 3). But still, the heterogeneity could not detect with these factors as none of them were not significant (Table 4).

**Table 3:** The pooled level of satisfaction and heterogeneity test by sample size and year of publication.

Grouping variable	Characteristics	Included studies	Effect size (95% CI)	Heterogeneity test (*I* ^2^)
Publication year	Before 2020	6	79.9% (67.2–92.5)	97.8%
	Since 2020	7	70.8% (64.4–77.2)	91.74%
Sample size	< 300 participants	8	77.4% (65.4–89.9)	98.03%
	> 300 participants	5	71% (63.8–78.3)	92.81%

**Table 4:** Factors related to the heterogeneity of included studies.

Variables	Coefficient	*p*
Publication year	1.58	0.209
Sample size	0.79	0.374

### 3.6 Sensitivity Analysis

**Figure 4:**
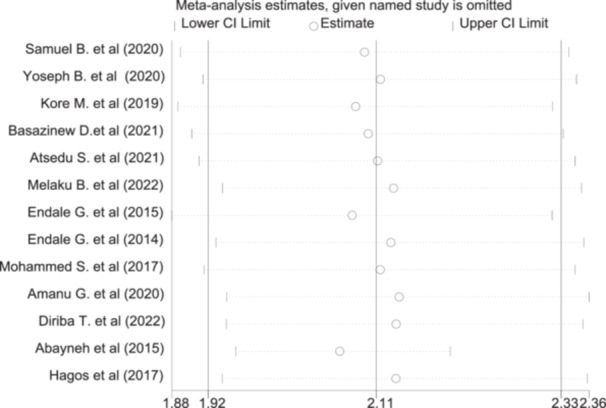
Sensitivity analysis of the level of satisfaction among patients who underwent surgery under anesthesia for anesthesia service in Ethiopia.

A sensitivity test was done using the random effect model and the result depicted that there was no single study that influenced the overall level of patient satisfaction significantly (Figure 4).

### 3.7 Factor Analysis

In this systematic review and meta‐analysis, seven studies were included to determine the pooled effect of factors that had an association with their primary studies. The factors detected are no postoperative nausea and vomiting, no postoperative pain, receiving premedication, type of anesthesia being regional anesthesia, having previous anesthesia exposure, and having pre and postoperative visits by anesthetists included in those seven studies. The analysis was done with a random effect model due to the heterogeneity of studies.

#### 3.7.1 Association of Postoperative Pain With Patient Satisfaction

**Figure 5:**
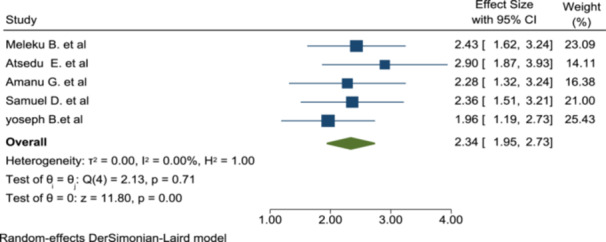
The association of postoperative pain with patient satisfaction of anesthesia service.

**Figure 6:**
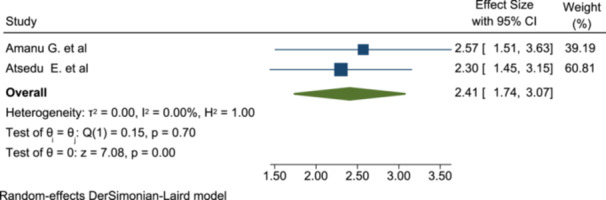
The association of PONV with patient satisfaction of anesthesia service.

In this meta‐analysis, five studies were included to do pooled effects of postoperative pain on patient satisfaction. As we see from Figure 6, postoperative pain is significantly associated with the level of patient satisfaction. Therefore, patients with no postoperative pain were around 2.34 times more satisfied in comparison with the patients with postoperative pain (OR: 2.34; 95% CI: 1.95–2.73) (Figure 5).

#### 3.7.2 Association of PONV With Patient Satisfaction

To determine the association of postoperative nausea and vomiting with the level of patient satisfaction, two studies were included in this meta‐analysis. PONV is significantly associated with patient satisfaction as patients with no PONV are more satisfied than their counterparts (OR: 2.41; 95% CI: 1.74–3.07) (Figure 6).

#### 3.7.3 Association of Regional Anesthesia With Patient Satisfaction

**Figure 7:**
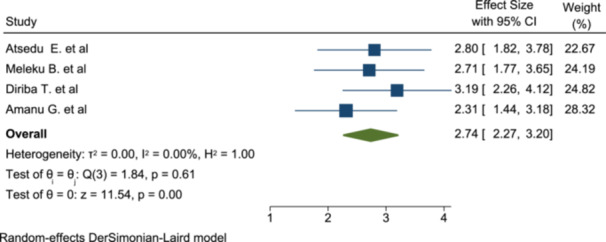
The association of types of anesthesia with patient satisfaction of anesthesia service.

Four studies were included to determine the pooled effect of types of anesthesia on patient satisfaction. Accordingly, patients who underwent surgery under regional anesthesia were 2.74 times more satisfied than patients who took general anesthesia (OR: 2.74, 95% CI: 2.27–3.2) (Figure 7).

#### 3.7.4 Association of Previous Anesthesia Exposure With Satisfaction

**Figure 8:**
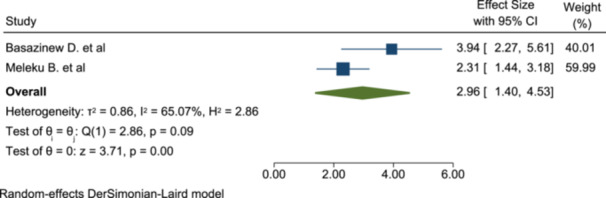
The association of previous anesthesia exposure with patient satisfaction of anesthesia service.

Based on the data extracted and analyzed from the two included studies, previous anesthesia history is significantly associated with the level of patient satisfaction. Patients having previous anesthesia exposure are more satisfied than their counterpart (OR: 2.96; 95% CI: 1.4–4.53) (Figure 8).

#### 3.7.5 Effects of Premedication on Patient Satisfaction

**Figure 9:**
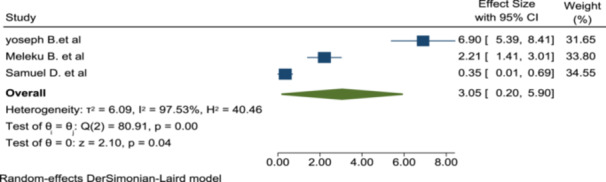
The association of premedication with patient satisfaction of anesthesia service.

Premedication given for nausea and vomiting prophylaxis, preoperative analgesia, and other possible premedication is assessed for its association with patient satisfaction. But the pooled effects of this factor are not significantly associated with satisfaction (OR: 3.05; 95% CI: 0.2–5.9) (Figure 9).

#### 3.7.6 Association of Perioperative Patient Visit by Qualified Anesthetist With Satisfaction

**Figure 10:**
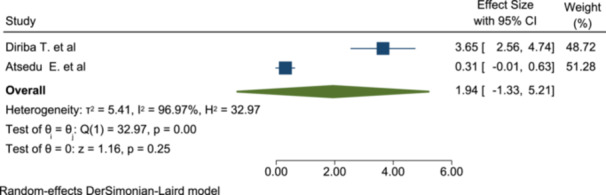
The association of perioperative patient visit by qualified anesthesia with patient satisfaction with anesthesia service.

Qualified anesthetists visit for the patient in the perioperative period is not significantly associated with patient satisfaction as done with data extracted from the two included studies (OR: 1.94; 95% CI: −1.33 to 5.21) (Figure 10).

## 4 Discussion

This systematic review and meta‐analysis was conducted to determine the pooled percentage of patient satisfaction and factors associated with anesthesia service in Ethiopia. From the included studies, the pooled percentage of patient satisfaction with anesthesia service is determined to be 75% with a 95% CI of 65%–84%. The proportion of satisfaction is high (79.9% (67.2%–92.5%) when the satisfaction was pooled from studies published before 2020 G.C., as compared with the pooled percentage (70.8% (64.4%–77.2%) of studies published after 2020 G.C. Also, the subgroup analysis showed that it was high for included studies, with a sample size of > 300 (77.4% (65.4%–89.9%) as compared with a sample size of < 300 (71% (63.8%–78.3%).

The main outcome of this study, the pooled percentage of patient satisfaction, is in line with the findings of the study done in Pakistan (76%) on similar topics [29].

The pooled level of satisfaction in this meta‐analysis is lower than studies done in Saudi Arabia and Kisumu Country Hospital (85%) [30, 31], in England by Oxford academicians (96.8%) [32], in Pakistan (95.2) [33], in Greece (96.3%–98.6%) [34], and in Korea, Ghana, and Thailand (96.3%) [8, 10, 35]. But it is higher than studies done in Eritrea (68.8%) [9]. These discrepancies could be explained by factors like sociodemographic characteristics of participants, sample size differences, and study time differences.

This systematic review and meta‐analysis detect factors that affect the pooled prevalence/level of patient satisfaction. These factors are postoperative pain, postoperative nausea and vomiting, type of anesthesia, previous anesthesia exposure, preoperative premedication, and pre‐ and/or postoperative anesthetist visits.

In this meta‐analysis, patients with no postoperative pain are 2.34 times more satisfied than their counterparts (OR: 2.34; 95% CI: 1.95–2.73). This is supported by studies done abroad. According to the studies done, patients having postoperative backache are more satisfied with anesthesia services [35, 36]. Also, patients who did not get adequate postoperative analgesia are more dissatisfied than those who got the analgesia [10]. Patients with complaints of perioperative pain are dissatisfied with the anesthesia service [29, 37].

Another factor that affects patient satisfaction significantly was PONV; patients who had no PONV were 2.4 times more satisfied than their counterparts (OR: 2.41; 95% CI: 1.74–3.07). This association is also explained by another study, which states that patients with PONV were more dissatisfied with anesthesia services [29, 35].

The patients who underwent the planned surgery with regional anesthesia, mainly spinal anesthesia, were more satisfied with the anesthesia service. It is around 2.74 times more satisfied (OR: 2.74, 95% CI: 2.27–3.2) than the patient who underwent surgery under general anesthesia. This is supported by the study done at Dr. Shariati's hospital and the University Hospital of America [38].

In this meta‐analysis, patients who had previous anesthesia exposure are more satisfied than those who were exposed for the first time. Those exposed previously are around three times more satisfied (OR: 2.96; 95% CI: 1.4–4.53). The possible explanation for this can be that patients who had previous exposure to anesthesia services might have information regarding their care during the perioperative period. Therefore, they become more satisfied with the repeated exposure.

## 5 Limitation of the Study

This systematic review and meta‐analysis was done by limiting the searched papers to English language only and which may limit as to get more studies that are published in the language other than English. Also there was a high level of heterogeneity among the included studies. Although we did meta‐regression and subgroup analysis with different factors and sensitivity analysis, the source of the variability could not be detected and resolved. As a solution, a random effect model, which is fitted for heterogeneous studies, was used for the analysis of the effect size. The other possible limitation of this work was that the discussion was compared with primary studies only.

## 6 Conclusion and Recommendation

According to this study, 75% of patients were satisfied with the anesthesia services they receive. Although this suggests a favorable judgment, there is still much room for improvement in perioperative care. The results showed that clinical comfort and the type of anesthetic method employed are the main factors influencing patient satisfaction. Particularly, the absence of postoperative pain and nausea/vomiting, the use of regional anesthesia, and prior anesthesia experience were the determinant factors. This nationwide evidence may be useful for the assessment of patient satisfaction and factors influencing satisfaction across the country and to compare it in low‐ and middle‐income countries. This finding might be helpful for policymakers and hospital administrators to give emphasis to the quality of perioperative anesthesia service and to improve the overall quality of perioperative patient care and outcomes. Also, the findings used for the caregivers including anesthesia professionals to be concerned on the factors affecting patient satisfaction like postoperative pain, postoperative nausea/vomiting, anesthesia type selection during anesthesia service. Therefore the community who will get the anesthesia service may get an advantage of quality care if the hospital administrators and clinical care give emphasis for this patient care outcome measures.

## Author Contributions

**Basazinew Chekol Demilew:** conceptualization, investigation, writing – original draft, methodology, writing – review and editing, software, formal analysis, data curation, supervision. **Agazhe Aemro:** methodology, writing – review and editing, supervision. **Habtie Bantider:** writing – review and editing, methodology, supervision. **Diriba Teshome:** methodology, writing – review and editing, software, data curation, supervision. All authors have read and approved the final version of the manuscript.

## Funding

The authors have nothing to report.

## Disclosure

Mr. Basazinew Chekol Demilew affirms that this manuscript is an honest, accurate, and transparent account of the study being reported; that no important aspects of the study have been omitted; and that any discrepancies from the study as planned.

## Ethics Statement

The authors have nothing to report.

## Consent

The authors have nothing to report.

## Conflicts of Interest

The authors declare no conflicts of interest.

## Supporting information

Supporting File

## Data Availability

The authors confirm that the data supporting the findings of this study are available within the article and/or its supporting materials. But if further necessary data will be required, we affirm that, it will be found from the corresponding author for a reasonable request. The corresponding author, Basazinew Chekol Demilew, had full access to all of the data in this study and takes complete responsibility for the integrity of the data and the accuracy of the data analysis.
